# The Role of Basolateral Amygdala and Medial Prefrontal Cortex in Fear: A Systematic Review

**DOI:** 10.7759/cureus.78198

**Published:** 2025-01-29

**Authors:** Volodymyr Mavrych, Fathima Riyas, Olena Bolgova

**Affiliations:** 1 Anatomy and Genetics, Alfaisal University College of Medicine, Riyadh, SAU

**Keywords:** animal models, basolateral amygdala, disease modeling, fear, medial prefrontal cortex

## Abstract

Fear is a primary adaptive response to potential threats. It triggers a complex cascade of physiological, cognitive, and behavioral changes that prepare an organism to cope with dangerous situations. The basolateral amygdala (BLA) and medial prefrontal cortex (mPFC) are both linked to adaptation, the generation of strong emotions, and decision-making. In this systematic review, we aimed to analyze recent studies of the connections between the BLA and mPFC in the context of their neuroanatomy, cellular composition, micro-circuitry, and involvement in fear. Utilizing the Preferred Reporting Items for Systematic Reviews and Meta-Analyses (PRISMA) 2020 guidelines, our search strategy involved scouring articles from PubMed (National Center for Biotechnology Information, Bethesda, Maryland), Google Scholar (Google, Mountain View, CA), and Science Direct (Elsevier, Amsterdam, Netherlands) databases covering the last decade (2014-2024). Thirty-two rigorously evaluated studies formed the essence of our review. Review findings revealed complex bidirectional connectivity between BLA and mPFC, with distinct roles for different subregions. The rostral BLA primarily projects to the prelimbic cortex, while the caudal BLA connects with the infralimbic cortex. These circuits show specialized cellular composition, with BLA containing principal excitatory neurons and GABAergic interneurons, while mPFC exhibits layer-specific synaptic connections. Fear processing involves dynamic interactions between these regions, with the prelimbic cortex promoting fear expression and the infralimbic cortex facilitating extinction. The analysis showed that astrocytic signaling and N-methyl-D-aspartate (NMDA) receptor activation are essential in the process of both fear memory formation and its extinction. There was evidence that dysregulation of specific circuits is associated with the pathophysiology of several other psychiatric disorders, such as post-traumatic stress disorder (PTSD), anxiety disorders, and schizophrenia. This review clarifies that the BLA-mPFC circuitry is critical in perceiving fear and its regulation. The results highlight the importance of the interactions between brain regions and the types of cells in each region to respond appropriately to fear and its extinction. Uncovering such type of dysregulation further helps to understand the mechanisms of fear-associated disorders and may suggest further treatment options. Future research should focus on cellular plasticity mechanisms, translational applications, and the influence of individual factors on fear processing to develop more effective treatments for psychiatric conditions involving fear dysregulation.

## Introduction and background

Fear is an evolutionarily primitive emotion that has affected the survival and development of animals in the entire animal kingdom. As a reaction to potential threats, fear activates a complex cascade of interrelated biological, mental, and behavioral changes that enable an organism to deal with perilous circumstances [1,2]. Stemming from evolutionary needs, the capacity of an organism to identify and act upon threats rapidly has been crucial for survival, and it led to the evolution of specialized neural circuits dedicated to the fear response [3,4].

Nonetheless, fear, like any other emotion, is a fundamental part of functioning, but it has some components that allow for dysfunction or disordered fear processes that can create disordered behaviors and can be predisposed to many psychiatric disorders like maladaptive variants, including specific phobias, panic disorder and post-traumatic stress disorder (PTSD) [2,5].

The neurobiology involved in generating fear is complex and incorporates various structures and transmitter systems within different brain regions. The basolateral amygdala (BLA) and the medial prefrontal cortex (mPFC) are two regions in this complexity's central part. These areas perform such functions and interact in such a way that they are crucial for learning, expressing, and regulating fear responses [6,7]. The amygdala, and in particular its basolateral complex, has been studied for a long time in conjunction with emotional processes, like fear and threat identification, among others [8]. Fears, reactions, and memories are all centralized in this consolidation hub [9,10]. The role of the BLA is not only about fears of reflexes but also about memories of emotional learning and decision-making processes that occur in threatening contexts [11,12].

The emotional mediators of the effect, such as fear, are still present with the contribution of the mPFC. Active emotional regulation is performed by the mPFC and its subdivisions (prelimbic (PL) and infralimbic (IL) cortices in rodents, dorsal anterior cingulate and ventromedial PFC in humans), which facilitate fear response as well as inhibit it, depending on the circuit involved. In this way, different fears can be adjusted depending on the information provided by the context, past experience, and the threat itself [13,14].

The interplay between the BLA and mPFC is a prominent circuit that enables dynamic modulation of fear. This circuit helps organisms acquire maladaptive fear, respond appropriately to stressful situations, suppress fear when it is not rational, and maintain appropriate fear behaviors as conditions change [15]. Knowledge about the roles of these regions and their connections is important for unraveling the basic neurobiology of emotion and better treating different disorders associated with fear [16,17].

Despite the numerous studies undertaken on the BLA and the mPFC regarding their role in fear, a systematic review of recent advancements in this area still needs to be completed. This review intends to fill this knowledge gap by analyzing available recent articles, looking at neuroanatomy, cellular composition, and micro-circuitry, and also computing the similarities and differences of BLA and mPFC areas due to the emotions of fear.

## Review

Materials and methods

This systematic review was conducted following the Preferred Reporting Items for Systematic Reviews and Meta-Analyses (PRISMA) 2020 updated protocol, ensuring standardized reporting of each stage of the research process, from literature search to data synthesis [18]. The participants, interventions, comparisons, outcomes, and study designs (PICOS) framework, which guides the review's objectives and scope, is outlined in Table 1.

**Table 1:** The PICOS framework utilized for the review's objectives and scope. PICOS: Participants; Interventions; Comparisons; Outcomes; Study Designs

PICOS	Description
P (Participants)	Animal models
I (Intervention)	Pharmacological, genetic, viral vectors, electrophysiological, optogenetic interventions and behavioral experiments
C (Comparisons)	Comparison of normal BLA-mPFC interaction in fear regulation with dysfunctional interactions in fear-related disorder animal models, or between different phases of fear processing.
O (Outcomes)	Synaptic/physiological changes and behavioral findings in animal models
S (Study Designs)	In vivo

Search Strategy and Information Sources

A systematic search strategy was employed with the intention of locating studies on the interaction of the BLA and the mPFC in the context of fear acquisition, fear expression, and fear extinction. The search involved three main databases: Pubmed (National Center for Biotechnology Information, Bethesda, MD), Google Scholar (Google, Mountain View, CA), and Science Direct (Elsevier, Amsterdam, Netherlands), which were best suited to cover the neuroscience literature.

The search query was formulated using a combination of keywords and Medical Subject Headings (MeSH) terms to maximize retrieval accuracy. The search terms included the following combinations: (Basolateral amygdala OR BLA) AND (Medial prefrontal cortex OR mPFC) AND (fear acquisition OR expression OR extinction). The search syntax, which combined these terms with Boolean operators (AND/OR), ensured retrieval of studies specifically addressing the BLA-mPFC interaction in fear processing. The search query was applied across titles, abstracts, and keywords. The systematic search targeting original peer-reviewed articles published in the last 10 years from September 24, 2014 to September 24, 2024 was completed and all citations were imported into Rayyan software (Rayyan Systems Inc., Cambridge, MA) for further processing, including duplicate removal and initial screening [19].

Eligibility Criteria

The inclusion and exclusion criteria for this review were rigorously defined to ensure only studies that met specific scientific standards were considered. Included studies had to be peer-reviewed original research articles, conducted in English, and focused on exploring the interaction between the BLA and mPFC. Additionally, the studies needed to examine the roles of the BLA and mPFC specifically in fear acquisition, expression, or extinction, and provide detailed information on neuroanatomy, connectivity, cellular composition, or functional dynamics within these neural structures. Conversely, studies were excluded if they lacked a direct focus on BLA-mPFC interaction, from non-peer-reviewed sources, review articles, conference abstracts, book chapters, dissertations, or focused on psychoactive drugs or psychostimulants like amphetamines, alcohol, or cocaine in fear processing contexts. Clinical studies involving human functional magnetic resonance imaging (fMRI) data and patient populations with neurological or psychological conditions beyond the typical scope of BLA-mPFC fear processing research were also excluded, as the review prioritized cellular and neuroanatomical findings.

Study Selection and Data Collection

The initial screening process was systematic; title screening was followed by abstract screening. The titles and abstracts of each article were carefully screened and compared to our set criteria for article inclusion and exclusion. As the first round of selection was completed, the second was started, which consisted of thoroughly reviewing the text of the articles selected previously. This stage was critical to further refine our selection based on the same inclusion and exclusion criteria applied earlier, with an emphasis on the rigor and relevance of the studies to our research objectives. Each full-text article was carefully reviewed to ensure that it not only focused on the interaction between the BLA and mPFC but also provided sufficient scientific depth regarding the mechanisms of fear processing being investigated. The rationale for these exclusions was to maintain the integrity and focus of the systematic review on studies that contribute meaningful insights into the neurobiological underpinnings of fear processing within the BLA-mPFC circuitry. This three-step screening process ensured that the final selection of studies included in the review would provide a robust and relevant dataset for understanding the complex interactions between these critical brain regions in the context of fear.

Data were extracted from each included study using a structured Microsoft Excel (Microsoft, Washington, DC) form to ensure a comprehensive capture of essential information. This form gathered details on study identification, including the first author, publication year, study title, and design. It also documented participant characteristics, such as the species used, sample size, age, and sex. Specific variables related to the BLA-mPFC interaction were recorded, covering neuroanatomical details, connectivity metrics, cellular compositions, and mechanisms of fear processing. Additionally, outcome data were collected, encompassing survival behaviors, learning metrics, social communication parameters, and motivational aspects linked to fear processing within the BLA-mPFC circuitry.

Bias Risk Assessment

In reviewing the included studies for possible risk of bias, we utilized the Systematic Review Centre for Laboratory Animal Experimentation (SYRCLE) (Nijmegen, Netherlands)risk bias assessment tool which was designed specifically for this purpose discerning the methodological quality and risk of bias of animal studies. This tool is grounded in the principles established by the SYRCLE and is designed to facilitate a comprehensive evaluation across various dimensions of study design [20].

During the assessment process, each study was systematically examined against a ten-question framework provided by the SYRCLE tool. The responses to these questions were categorized as “YES” (indicating minimal risk of bias), “NO” (indicating significant risk of bias), or “UNCLEAR” (denoting uncertainty regarding the potential for bias). This categorization helped in identifying the strengths and weaknesses of each study, facilitating an overall understanding of the reliability of the results presented in the context of BLA-mPFC interactions in fear processing.

Results

**Figure 1:**
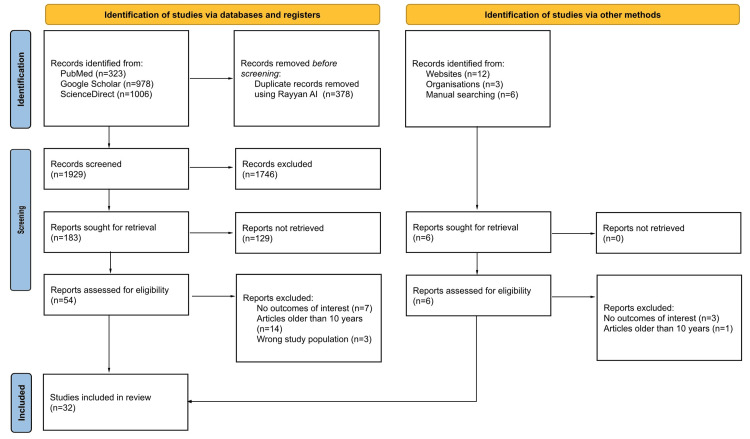
The PRISMA diagram demonstrates the detailed search strategy, which was utilized in this systematic review. PRISMA - Preferred Reporting Items for Systematic Reviews and Meta-Analyses

From our search in PubMed, Google Scholar, and Science Direct databases, we received 2,307 articles, which were imported into Rayyan software. Upon importing the records, Rayyan automatically identified and removed 378 duplicate entries. Exploration through manual search yielded an additional 21 records. Following this, a preliminary assessment of titles and abstracts was performed, and 1,890 studies were excluded out of 1,950 due to irrelevance to the core objectives of the review. Subsequently, 60 articles progressed to full-text review, from which a further 28 were eliminated due to the lack of adherence to the inclusion criteria. Ultimately, 32 studies met the inclusion criteria in this systematic review. To depict the multi-stage review process, and increase methodological transparency, a presentation of studies selection was done using a PRISMA flowchart showing the stages starting from the initial search to the last studies that were considered in the review (Figure 1). This selection process, in turn, contributed to the rigorousness and transparency of the methodology making it easy to grasp the complex relationships between the BLA and the mPFC in relation to fear.

All 32 core studies included in this review utilized animal models: 14 focused exclusively on rats, 15 on mice, one included both cats and rats, one focused on monkeys, and one on guinea pigs. Sample sizes varied widely, ranging from four to 208 animals per study, and most of the studies (except three) used only male animals from postnatal day 3 to adulthood developmental stages. These studies targeted the neural mechanisms responsible for fear processing, memory, and extinction, focusing specifically on the BLA and the mPFC. Pharmacological, genetic, viral vectors, optogenetic, and behavioral trials provide knowledge of how these events are mediated by brain structures.

**Figure 2:**
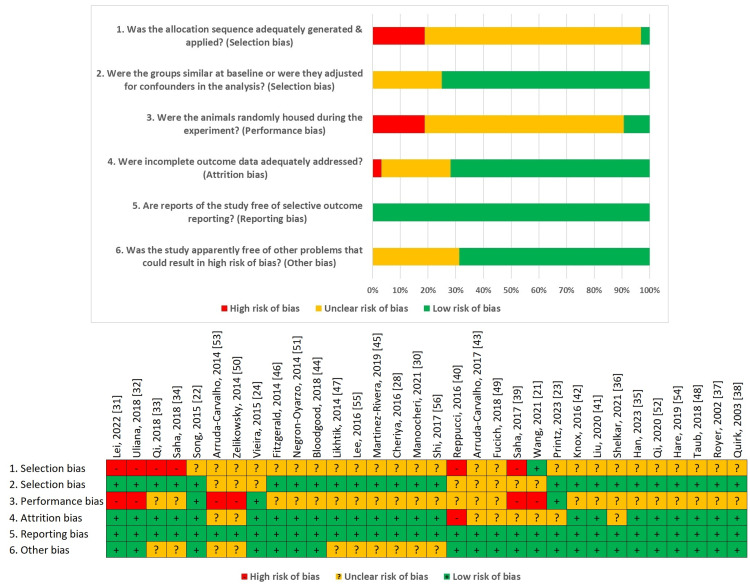
Qualitative analysis with SYRCLE’s risk of bias for 32 animal studies demonstrates the distribution of high, unclear and low risk of bias. SYRCLE - Systematic Review Centre for Laboratory Animal Experimentation Ref. [20]

To assess the risk of bias in the included studies, we used the SYRCLE tool [20]. It categorizes responses as “YES” (indicating low risk of bias), “NO” (indicating high risk of bias), or “UNCLEAR” (indicating insufficient information to assess bias risk). Our findings are summarized in Figure 2, and they reveal that certain bias domains were consistently problematic across many studies.

One notable area of concern involved the criterion, “Was the allocation sequence adequately generated and applied?” However, a large number of studies did not seem to satisfy this criterion, with only one study [21] having received a “YES” rating suggesting that a lot of studies are likely to have selection biases due to poor allocation procedures. Regarding baseline comparability, the criterion “Were the groups similar at baseline or were confounders adjusted for in the analysis?” was generally well-addressed, with most studies receiving a “YES” rating, indicating a low risk of bias in this area. However, several studies were marked as “UNCLEAR,” reflecting insufficient information to confirm baseline equivalence or adjustment for confounders. The criterion assessing random housing of animals, “Were the animals randomly housed during the experiment?” showed mixed results. A few studies [22-24] met this criterion with a “YES” rating, indicating a low risk of bias. However, many studies received a “NO” or “UNCLEAR” rating, indicating a potential bias risk due to non-random housing conditions. Most studies performed well in handling incomplete outcome data, with a “YES” response indicating a low risk of bias. Selective outcome reporting was generally minimal across studies, as the majority were rated as “YES” for the criterion, “Are reports of the study free of selective outcome reporting?” This finding suggests a low risk of bias related to selective reporting. Finally, for the criterion assessing other potential sources of bias, “Was the study free of other problems that could result in a high risk of bias?,” most studies were rated as “YES,” suggesting a low risk of bias in this domain. However, some studies were rated “UNCLEAR,” indicating potential, but unspecified, sources of bias.

In summary, it was noted that although the overall risk of bias was low in most domains, some concerns were raised regarding the generation of allocation sequence applied and random housing of animals. These conclusions highlight the need to improve methodological rigor in subsequent investigations in order to reduce bias and enhance the validity of the obtained results.

Discussion and future directions

Our review systematically examines recent original research based on different animal models to clarify the relationship between the BLA and mPFC in the context of their neuroanatomy, cellular composition, micro-circuitry, and involvement in emotion of fear. Utilizing diverse methods, from chemogenetic activation of specific receptors to optogenetics, these studies indicated significant connections between BLA and mPFC, notably linked to different anxiety disorders, PTSD, and schizophrenia. Table 2 (Appendix) provides in-depth information on the specifics of each study, including animal investigation, model generation strategies, behavioral testing, key findings regarding animal behavior, and related disorders.

Neuroanatomy and Connectivity

Many aspects of the interrelation between the mPFC and the BLA have remained highly contentious within the field of neuroscience. One major controversy is about the hierarchical organization of these structures. The traditional view, championed by LeDoux, Šimić, and Sun, suggests that the amygdala processes emotional information first and then feeds this information to the mPFC for higher-order regulation [25-27]. However, recent studies using optogenetic approaches [28-30] have revealed a more complex picture, suggesting a distributed and recursive processing model.

Another contentious area concerns the specificity of BLA-mPFC pathways. While some researchers argue for distinct, functionally segregated circuits [28,30], others support a more integrated model where individual neurons participate in multiple functional networks [23]. This debate has significant implications for understanding emotional regulation and psychiatric disorders.

The amygdala is an almond-shaped structure found deep in the temporal lobes and is involved in emotional processing, especially fear [31,32]. The amygdala's basolateral complex (BLA) is specifically involved in fear learning and expression [33,34]. BLA includes the lateral nucleus (LA), basal nucleus (BA), and accessory basal (AB) nucleus, which serves individually distinct connectivity and functional roles within the framework of fear processing.

It has been reported that the connectivity of BLA is quite organized and can be divided into two distinct parts based on the cortical areas they project to. The rostral BLA (rBLA) predominantly innervates the PL cortex, while the caudal BLA (cBLA) innervates the IL cortex [28,30]. These projections are organized topographically and separately influence different brain regions. BLA is one of the structures in the circuit that encompasses the mPFC, hippocampus, and other regions such as the bed nucleus of stria terminalis (BNST), central nucleus of the amygdala (CeA), and periaqueductal gray (PAG) [28]. This network is crucial for the management of emotions as well as behaviors that are instigated by fear [35].

Despite significant advances in our understanding, several crucial knowledge gaps persist in the field. More detailed knowledge of specific interneuron types and BLA-mPFC communication is needed, but a satisfactory explanation has yet to be provided. Another uncertain aspect of the BLA-mPFC circuits is their development progression and the critical timeframe needed for full maturation. Moreover, the mechanisms that underlie the individual variability in the BLA-mPFC circuit and the functional role in the emotional circuitry still need to be better understood. Understanding these variations, however, may present some clues as to why there are individuals who are more prone to emotional disorders than others.

Looking into the future perspective, a number of advances are likely to enhance the current understanding of the BLA-mPFC circuits. Viral tracing tools should become an increasingly effective means of determining the subspecification of the BLA and its mPFC targets. Real-time circuit analysis through technologies like fiber photometry and miniscope imaging will enable a better understanding of how these circuits function during natural behavior. These advances and sophisticated computational approaches may help integrate diverse findings into coherent mechanistic models of BLA-mPFC function.

Cellular Composition and Micro-circuitry

The cellular composition and micro-circuitry of the BLA are complex, with astrocytes playing a key role in synaptic transmission, neuronal excitability, and memory processes [31,36]. The BLA consists of principal excitatory neurons and GABAergic interneurons, with the latter comprising approximately 10%-15% of the neuronal population [34]. The amygdala's intercalated (ITC) neurons serve as gatekeepers of fear responses. They regulate fear expression by controlling the flow of signals between the BLA, which receives fear-related inputs, and the central nucleus (Ce), which generates fear responses [37,38].

GABAergic synapses, particularly at the axon initial segment of projection neurons, are crucial for modulating fear extinction [34,39]. BLA projection neurons show differential excitability based on fear learning paradigms [22] and project to various regions, including the nucleus accumbens. These projections evoke both excitatory and inhibitory responses, with stronger excitatory connections to layer 5 cortico-PAG neurons in the IL cortex [28]. BLA exhibits specialized cellular circuits where different neuronal populations innervate the mPFC and LHA, forming parallel processing pathways to regulate emotional and motivated behaviors [28,30,40].

Stress can significantly impact BLA structure and function: chronic stress enhances dendritic length and spine quantity, increasing firing rate and membrane excitability [41]. Single-prolonged stress (SPS) can induce apoptosis in BLA, particularly affecting inhibitory GABAergic neurons, which disrupts fear extinction processes [42].

The mPFC's cellular composition and microcircuitry facilitate fear processing and emotional regulation [37,38]. Two main types of neurons are identified: Regular Spiking Neurons, which exhibit consistent firing patterns and are involved in fear learning and extinction, and Burst Spiking Neurons, which fire in high-frequency bursts associated with synaptic plasticity and fear acquisition [22,43].

Layer-specific synaptic connections exist between the BLA and neurons in the mPFC (particularly in layer 5) [28]. The BLA-mPFC circuit includes both GABAergic and glutamatergic projections, with GABAergic projections significantly affecting mPFC plasticity [34]. Structural changes in the mPFC, including spine loss and dendritic retraction, occur under stress conditions [41]. Single prolonged stress (SPS) can reduce excitatory tone and induce apoptosis in the mPFC, particularly in the IL region, affecting its ability to inhibit the BLA during extinction [42].

Our understanding of cell-type specificity still needs to be completed. Despite knowing the major cell types, we lack a detailed comprehension of molecular diversity within principal neuron populations, complete interneuron taxonomy, and cell-type-specific connectivity patterns. Circuit dynamics present another significant gap, with limited knowledge regarding real-time circuit operations during behavior, mechanisms of synaptic plasticity in specific pathways, and integration of multiple inputs at the cellular level. We need to understand circuit formation during development, critical periods for circuit refinement, and the impact of early life stress on circuit maturation.

Technological advances are expected to drive progress in several areas. New tools, including enhanced genetic targeting methods, improved voltage indicators, novel viral tracers for circuit mapping, and advanced microscopy techniques, will enable better investigation of these circuits. Analytical methods are also advancing, with machine learning approaches for behavior analysis, advanced circuit function computational models, and better multi-modal data integration.

Fear Processing

BLA and mPFC play a central role in fear processing, with astrocytes modulating BLA-mPFC communication crucial for fear memory processing [31,36]. The mPFC integrates sensory inputs and emotional cues from the BLA, coordinating both the expression and inhibition of fear responses depending on the environmental context [35]. These connections undergo significant changes during adolescence, which may influence fear processing [43].

The PL and IL regions of the mPFC serve distinct functions in fear processing. The PL is associated with fear maintenance and retrieval, while the IL is involved in fear extinction [44,45]. This balance between PL (fear expression) and IL (fear inhibition) activity determines whether fear responses are sustained or inhibited [46].

The BLA-mPFC pathway shows distinct activity patterns during fear acquisition and extinction, with long-term potentiation (LTP) being essential for fear extinction [37]. Different patterns of neural excitability are observed in BLA projection neurons during various phases of trace fear conditioning [22]. The structure processes fear signals through multiple mechanisms: theta synchrony with the mPFC helps distinguish between safety and threat [35,47], while projections to both the mPFC and lateral hypothalamus (LHA) control different aspects of fear and motivated behavior through parallel pathways [28,40].

Fear processing in the BLA involves integrating excitatory neurotransmission with N-methyl-D-aspartate (NMDA) receptors (particularly GluN2C subunits), enhancing synaptic plasticity crucial for fear learning [36]. The BLA processes fear-related information by dynamically switching between oscillatory states: low theta band oscillations link to freezing, while high theta and beta bands associate with flight [35]. The development of synaptic transmission between mPFC and BLA contributes to memory specificity and generalization [21,43].

Chronic stress can alter the fear processing circuitry, notably through enhanced glutamatergic transmission from the mPFC to the BLA, leading to exaggerated fear responses. However, the mPFC's involvement in processing fear and regulating emotional responses is central to reversing stress-induced impairments and restoring cognitive flexibility [41].

Acquisition of Fear Memories

The acquisition of fear memories heavily involves BLA, with astrocytic activation during fear conditioning enhancing auditory-cued fear memory formation [31]. During fear conditioning, sensory information about the unconditioned stimulus (US) and conditioned stimulus (CS) converges in the BLA, leading to the storage of CS-US associations [48,49]. This process involves NMDA receptor activation and synaptic plasticity, which is crucial for fear memory formation [24].

The BLA's bidirectional connections with the mPFC are critical for this process [28,30], with specific pathways serving different functions: rBLA projections to PL are involved in threat conditioning expression, while cBLA projections to IL relate to threat response extinction [30]. The development and maturation of mPFC-BLA circuits coincide with increased synaptic activity [43], suggesting early participation in fear memory encoding. The BLA strongly excites back-projecting mPFC neurons, likely facilitating fear memory acquisition [23].

Fear Expression and Maintenance of Learned Fear

The reciprocal connectivity between the BLA and mPFC creates a streamlined communication loop for processing fear-related information [28,30]. BLA activity is particularly critical for conditioned fear expression, with its functional connectivity modulating contextual fear expression during extinction testing [42].

The expression of fear involves complex interactions within the BLA, with chemogenetic activation of BLA astrocytes enhancing fear memory acquisition but not expression [31]. BLA neurons that encode initial fear memories are reactivated during fear expression [50], with increased neuronal activity in the BLA activating the CeA during fear recall [38]. BLA manipulation, such as neurofascin knockdown, can impair fear expression through reduced GABAergic synapses, affecting BLA-mPFC plasticity [34,39].

The PL region of the mPFC plays a crucial role in fear expression, receiving inputs from the BLA that enhance fear responses [45]. Avoiding memory retrieval is associated with increased activation in PL neurons projecting to BLA [45]. The reciprocal connections between mPFC and BLA are vital for fear expression and maintenance, with changes in these synapses affecting the ability to process and maintain fear responses [21,28]. The development of mPFC projections to the BLA supports the maturation of circuits involved in fear expression [43].

The BLA exhibits synchronized activity with the mPFC, especially in the low theta band (3-7 Hz), which plays a causal role in driving fear-related behaviors like freezing [35]. This process is modulated by BLA astrocytes and NMDA receptors, which influence both fear conditioning and extinction processes through the regulation of synaptic plasticity [36].

Fear Extinction and Safety Signaling

The mPFC plays a crucial role in fear extinction and safety signaling, with BLA-to-mPFC projections being particularly important in modulating these processes [44]. The IL region of the mPFC is especially critical for fear extinction and safety signaling [36,44]. ITC neurons in the amygdala can undergo NMDA-dependent synaptic changes (LTP and LTD), which are suggested to play a role in the extinction of fear responses [37] and activation of astrocytes in the BLA-facilitated fear extinction, resulting in reduced freezing behavior during the post-extinction recall test [36].

Extinction training produces significant changes in neural activity, including reduced excitability of mPFC neurons in the PL area and increased excitability of IL-BLA projection neurons [22,44]. The IL cortex projects to the amygdala's GABAergic ITC cells and central nucleus, which are involved in fear inhibition [28,37]. This circuit is crucial for distinguishing between threatening and non-threatening stimuli. Single prolonged stress can attenuate the IL's ability to enhance its neural activity during extinction, leading to impaired extinction retention [42].

The mPFC-BLA circuitry is essential for the extinction of fear responses and safety signaling [49], with cBLA projections to IL being specifically implicated in the extinction of threat responses [30]. This interaction helps encode safety signals that diminish learned fear responses over time. Therapeutic interventions that activate the mPFC, particularly its ventromedial region, have been shown to facilitate fear suppression and enhance the re-extinction of conditioned fear [49].

Contextual Modulation of Fear Responses

The mPFC plays a crucial role in the contextual modulation of fear responses, with communication between the BLA and mPFC essential for this process [47]. The mPFC's PL cortex and IL cortex work together to integrate context recognition and its association with potential threats [45,49]. This integration allows the brain to differentiate between safe and dangerous environments [35].

The system functions through a complex network involving the hippocampus, which provides critical input to the PL for distinguishing between danger and safety during extinction testing [42]. This three-way interaction between mPFC, BLA, and hippocampus is crucial for contextual aspects of fear learning [50].

The mPFC's ability to contextualize fear responses is essential for adaptive behavior [45]. It processes safety signals that help reduce generalized fear [24], and disruptions in the mPFC-BLA circuit can lead to overgeneralization of fear in non-threatening contexts [32]. The distinct activation patterns within the PL and IL by different BLA projections suggest specialized roles in modulating fear responses depending on the context [28,30]. The mPFC dynamically adjusts its activity to regulate freezing and flight behaviors in response to threats [35], with stress potentially disrupting this modulation. However, therapeutic interventions can help restore proper mPFC function [49].

Fear Generalization

The BLA plays a significant role in fear generalization, with evidence suggesting that astrocytes in the BLA contribute to this process through their effects on communication with the mPFC [31]. Hyperactivity in the BLA is linked to generalized anxiety and fear disorders such as PTSD, where overgeneralization of fear responses occurs due to malfunctioning fear inhibition circuits [24]. The PL cortex, modulated by BLA input, plays a role in generalizing fear responses based on sensory and emotional cues [45].

Single Prolonged Stress (SPS) can dysregulate the BLA, affecting fear generalization by altering connectivity with the hippocampus, which is essential for distinguishing between fear and extinction contexts [42]. Fear generalization may also be influenced by NMDA receptor activity [24], although specific pathways for generalization versus extinction are distinct.

The BLA functions as part of a network that enables switching between behaviors based on threat context. This allows for flexible generalization of responses, such as freezing or flight, depending on the situation [35].

Emotional Regulation

The mPFC plays a central role in emotional regulation, particularly through its interactions with the BLA. Dysregulated astrocytic signaling in this circuitry may contribute to emotional dysregulation in fear-related disorders [31,36].

The IL cortex specifically modulates emotional regulation by dampening fear responses through projections to inhibitory neurons in the amygdala [37,44]. This system coordinates between higher cognitive functions and lower emotional responses, with dysregulation in this pathway being implicated in disorders such as anxiety and PTSD [42].

The mPFC-BLA circuitry balances excitatory inputs and fine-tunes emotional output related to fear [28,30]. This interaction involves dynamic oscillatory activities, including theta and beta rhythms, which play a critical role in coordinating defensive behaviors like freezing or flight [35]. The system is particularly important for behavioral flexibility and autonomic responses to stress [49]. Stress-induced changes in this regulation are linked to the development of anxiety disorders [51], with the mPFC working to suppress fear under non-threatening conditions.

Through its complex connectivity with the BLA and other brain regions, the mPFC ensures appropriate emotional responses by modulating amygdala output [38]. Dysfunction in this circuit could contribute to the pathology of fear-related disorders by disrupting oscillatory patterns and impairing emotional regulation [52].

Dysregulation in Fear-Related Disorders

The dysregulation of BLA functions, particularly in BLA-to-mPFC projections and astrocytic signaling, plays a significant role in various fear-related disorders [31]. BLA shows hyperactivity in several clinical conditions: in the methylazoxymethanol acetate (MAM) model of schizophrenia [32], in stress-related psychiatric disorders [41], and PTSD [42]. This hyperactivity can lead to persistent fear memories and maladaptive fear responses. Dysregulation manifests through multiple mechanisms: abnormal GABAergic synapses impair fear extinction [34,39], chronic stress affects dendritic morphology [53], and abnormal BLA connectivity with PL and IL cortices leads to persistent fear responses and impaired fear extinction [51].

The impairment of mPFC can manifest in multiple ways: reduced activity during fear conditioning (as seen in schizophrenia models) [32], disrupted fear extinction from chronic stress, and difficulties in behavioral flexibility [34]. This dysfunction is particularly evident in the balance between the PL and IL areas, where dysregulation can result in impaired fear extinction [44]. In PTSD cases, reduced IL activity hinders patients' ability to suppress fear after the danger has passed, while PL overactivation leads to excessive fear expression [42].

The BLA-mPFC connectivity dysfunction is implicated in various psychiatric conditions, including anxiety disorders and depression [52,54]. Hare and colleagues found that selectively activating neurons containing D1 dopamine receptors (Drd1) in mPFC quickly reduced symptoms of depression and anxiety in animals, with effects lasting well beyond the stimulation period. In contrast, when they activated neurons containing D2 dopamine receptors (Drd2), they observed no such improvements [54].

In PTSD models, stress enhances BLA activity, contributing to persistent fear expression and extinction retention deficits [42,55]. Dysregulation can also manifest as inappropriate synchrony or failure to switch between defensive responses (freeze versus flight), contributing to maladaptive fear responses [35]. These disruptions in reciprocal circuits affect the balance of excitatory and inhibitory inputs [55].

Therapeutic approaches focusing on enhancing mPFC function have been proposed as potential treatments for these conditions, with extinction learning and pharmacological interventions targeting the mPFC to alleviate these dysfunctions [49,56]. At the molecular level, dysregulation involves malfunctioning NMDA receptor pathways in the BLA [24,56], so some studies suggest that enhancing astrocytic NMDA receptor function could improve fear extinction, offering potential therapeutic targets [36].

Despite significant progress, several crucial knowledge gaps persist. There is a considerable research gap that addresses the individual aspects of fear processing and resilience. Why do some individuals develop anxiety disorders and others do not, even when exposed to the same threats? The influence of environmental and genetic factors on BLA-mPFC circuit function also needs to be examined. Another unexplored area is the disassociation between conscious and unconscious fear processing. How are these circuits involved in processing and integrating explicit and implicit threat information, and how are they combined? This question has significant consequences for understanding and treating anxiety disorders where conscious and unconscious fears are likely to be disassociated.

Limitations

Despite the structured search strategy in multiple databases and the specific eligibility criteria and goals, this review has limitations. First, we focused on the relationship between BLA and mPFC and did not include detailed evaluations of other brain regions, such as vHipp, BNST, CeA, and PAG. Second, we did not perform a literature analysis of studies on humans. 

We set the time limit to the last 10 years in our search criteria, and only publications in English were considered, which may have resulted in missing some studies. It is also possible that the method used, using only peer-reviewed academic papers, yielded only those studies that were in conformity with the results generalized in the literature. As a result, this may lead to an overestimation of BLA-mPFC relationships.

Finally, this review only considers studies with direct brain measurements, neglecting that stress and emotional aspects could also be assessed stress and emotional aspects indirectly. Additionally, the reviewed studies also have limitations. While analyzing the data, we identified gender (more males) and age (preference for the young) imbalance, and few studies had a longitudinal design (limiting causality inference). Another limitation often found was the short exposure time for the tested conditions.

Future research

One important direction in which research should proceed is related to cellular and subcellular plasticity that facilitates the encoding of fear memories. In particular, studies should investigate epigenetic change mechanisms during memory trace consolidation, protein synthesis requirements, and neurotrophic signaling in both areas. Some fundamental aspects of fear memory acquisition may be explained by a thorough comprehension of dendritic spine dynamics and synaptic rearrangements in the course of fear conditioning and, afterward, stress responses. These principles may come in handy for developing effective treatment approaches for different disorders that contain fear components.

Translational research is the second important direction, which concerns applying the basic findings to practice. This includes creating circuit-specific pathological fear state biomarkers and developing other imaging protocols for assessing circuit integrity. Explaining the depreciation of symptoms in relation to the areas of the circuit actively moderated within the scope of practice would enable better diagnosis of disorders. Moreover, applying circuit-specific neuromodulation methods and designing specific medications might transform the management of fear-related disorders.

To accomplish a fuller picture, the role of individual and external factors in fear processing should be examined in great detail. Further, it would be pertinent to study the effects of genetic polymorphisms on the functioning of circuits, the effects of early-age stressors on the development of circuits, and the effects of environmental biofeedback on acquiring the threat. Grasping these processes could clarify why some individuals are more anxious than others and why some respond differently to fear-inducing situations.

Finally, connectivity between other regions of the brain and computational aspects is very important. Studies need to include mapping the ectopic site connections with the hippocampus and those with the sensory areas, research of the dopamine reward system interaction with other pathways, and research of the autonomic system interaction. Such studies could also involve the creation of artificial networks for data analysis and the building of models of circuit dynamics. This understanding at this level may help develop new therapeutic strategies for patients with fear-related disorders.

## Conclusions

This systematic review examines the connections between BLA and mPFC and a range of neuropsychiatric disorders, employing an approach of animal models. The review highlights the significant advances in understanding BLA-mPFC interactions, particularly in anxiety, PTSD, GAD, and schizophrenia. New therapeutic approaches might combine traditional behavioral treatments with precise neuromodulation techniques in further developments.

Additionally, the review underscores the importance of integrating increasingly complex datasets into coherent models of fear processing. Success will require combining insights from molecular, cellular, circuit, and behavioral levels of analysis. While many questions remain, continued investigation of BLA-mPFC interactions promises to advance both our basic understanding of emotion and our ability to treat fear-related disorders.

## Appendices

**Table 2:** Overview of recent animal-based studies detailing animal use, model generation, behavioral testing, main findings, and correlated disorders. AAV - Adeno-Associated Virus Vector; AFC - Auditory Fear Conditioning; AFD - Auditory Fear Discrimination; AST - Attentional Set-Shifting Test; BLA - Basolateral Amygdala; CEA - Central Amygdala; CFT - Contextual Fear Test; CNO - Clozapine-N-oxide; CPP - Conditioned Place Preference; CR - Conditioned Response; CRS - Chronic Restraint Stress; CUMS - Chronic Unpredictable Medium Stress; dACC - Dorsal Anterior Cingulate Cortex; DFC - Differential Fear Conditioning Task; DLB - Dark-Light Box Test; EGD - Explore and Groom Detection; EPM - Elevated Plus Maze; ERT - Extinction Recall Test; ET - Extinction Training; EXT - Extinction Test; EZM - Elevated Zero Maze; FC - Fear Conditioning; FCEP - Fear Conditioning and Extinction Paradigm; FE - Fear Extinction; FFD - Freeze and Flight Detection; FS - Foot Shock; FST - Forced Swim Test; GAD - Generalized Anxiety Disorder; IL - Infralimbic Cortex; ISD - Immediate Shock Deficit; ITC - Intercalated Cells; LAT - Locomotor Activity Test; LTD - Long-Term Depression; LTM - Long-Term Memory Test; LTP - Long-Term Potentiation; MAM - Methylazoxymethanol Acetate; MBT - Marble-Burying Test; mPFC - Medial Prefrontal Cortex; NAPR - Non-Associative Place Recognition; NMDAR - N-methyl-D-aspartate Receptors; NSF - Novelty Suppressed Feeding; OFT - Open Field Test; PAG - Periaqueductal Gray; PERT - Post-Extinction Recall Test; PL - Prelimbic Cortex; PMAT - Platform-Mediated Avoidance Task; PPF - Paired-Pulse Facilitation; PTSD - Post-Traumatic Stress Disorder; rBLA/cBLA - Rostral and Caudal Basolateral Amygdala; Ret - Retrieval; SI - Social Interaction Test; SPDB - Shock-Probe Defensive Burying Test; SPS - Single Prolonged Stress; SPT - Sucrose Preference Test; TC - Threat Conditioning; TER - Tone-Evoked Retrieval; TFC - Trace Fear Conditioning; VS - Ventral Striatum; WM - Water Maze Reversal Learning Task

References	Animal use	Model generation	*Behavioral Testing	Main findings	Correlated disorders
Lei, 2022 [31]	Mice	AAV injection to deliver hM3Dq receptors into BLA astrocytes, CNO administration	FC, NAPR, OFT, EZM	Astrocyte Gq activation enhances fear memory, no effect on mild stress memory/anxiety, no impact during memory retrieval, increases c-Fos in BLA/mPFC during conditioning	Anxiety, PTSD
Uliana, 2018 [32]	Rats	MAM conditioning, in vivo extracellular recordings of BLA-evoked responses in mPFC, intra-BLA injection of an NMDAR antagonist (AP5)	FS, FC	MAM model impairs tone fear extinction, normal contextual fear conditioning, Disrupted BLA-mPFC interaction, BLA glutamate transmission deficits, lack of fear extinction	Schizophrenia
Qi, 2018 [33]	Mice	Resident-intruder social defeat paradigm, muscimol (GABA agonist) microinjections into BLA, immunohistochemistry, gene expression analysis (zif268, arc, c-Fos)	OFT, SI, SPT, MBT	Social fear memory lasts 7 days, BLA activity links to social fear formation, vHIP/mPFC show initial suppression then recovery, BLA muscimol reduces fear and increases vHIP activity	Chronic social defeat, anxiety
Saha, 2018 [34]	Rats	Lentiviral neurofascin (NF) gene knockdown in BLA, BLA-mPFC pathway and ventral subiculum-mPFC synaptic plasticity recordings	OFT, EPM, WM, FC, FE	BLA neurofascin loss impairs fear extinction, no effect on general anxiety, impaired reversal learning/flexibility, reduced BLA-mPFC synaptic plasticity	Cognitive flexibility impairments
Song, 2015 [22]	Rats	BLA retrograde fluorescent tracer infusion, mPFC-BLA neuron patch-clamp recordings	TFC, EXT	Trace fear conditioned group shows high post-CS freezing, extinction training reduces freezing, fear memory persists 11 days without extinction	PTSD, anxiety
Arruda-Carvalho, 2014 [53]	Mice	ChR2-AAV viral injections in PL/IL, optical stimulation of mPFC-BLA circuits, BLA neuron patch-clamp recordings in PL and IL pathways	FC, TER, CFT	CS triggers increased freezing, PL-BLA pathway shows plasticity changes, enhanced AMPA receptor function, altered excitation balance	Anxiety, fear-related disorders
Zelikowsky, 2014 [50]	Rats	catFISH for Arc mRNA detection, BLA/DH/mPFC activity analysis	CFC, ISD	Delayed shock causes fear conditioning, immediate shock prevents conditioning, dorsal hippocampus active during all context exposure encodes spatial information, BLA active with fear memory encodes emotional significance	PTSD
Vieira, 2015 [24]	Mice	Grin1 gene (NR1 subunit of NMDA) conditional deletion in mPFC CaMKIIa-Cre expression system	AFD, FC, FE	mPFC NMDAR loss impairs CS+/CS, discrimination, animals show generalized fear response and impaired fear extinction, NMDARs crucial for fear regulation	PTSD, fear-related disorders
Fitzgerald, 2014 [46]	Mice	Picrotoxin infusion, fluoxetine treatment, PL/IL mPFC single-unit recordings	FC, ET, ER	Poor extinction links to increased PL firing, IL activation alone can't suppress fear, PL/IL imbalance impairs extinction	PTSD
Negron-Oyarzo, 2014 [51]	Rats	7-day restraint stress, electrophysiological recordings	OFT, EPM, DLB, FCEP	Chronic stress in adolescence slows fear extinction and enhances anxiety-like behavior, stress-induced changes in PL and learned fear extinction were reversed by adulthood	Anxiety
Bloodgood, 2018 [44]	Mice	IL-BLA and IL-NAc chemogenetic manipulations, electrophysiological recordings	FCEP	IL-BLA pathway crucial for fear extinction, extinction increases IL-BLA neuron excitability, IL-BLA inhibition impairs fear extinction recall	PTSD
Likhtik, 2014 [47]	Mice	mPFC/BLA/hippocampus LFP recordings	DFC, OF	Successful discrimination between safe and aversive cues shows increased mPFC-BLA theta synchrony, theta entrainment of BLA activity to mPFC signals safety and reduced fear	PTSD, GAD
Lee, 2016 [55]	Rats	Arc catFISH mRNA detection, mPFC/amygdala activity analysis	FC, Ret, FE	Extinction and retrieval + extinction led to different behavioral and neural outcomes, retrieval + extinction reduces fear responses and helps prevent the return of fear	PTSD
Martinez-Rivera, 2019 [45]	Rats	BLA/VS retrograde tracer infusions, c-Fos activity analysis	PMAT, Ret, Ext	Retrieval of avoidance activated PL neurons projecting to the BLA but not to the VS, extinction activated IL neurons projecting to both BLA and VS, and PL neurons projecting to VS	Anxiety
Cheriyan, 2016 [28]	Mice	BLA-PL/IL pathway identification, optogenetic stimulation, synaptic response recordings		BLA shows region- and laminar-specific targeting within PL and IL regions of mPFC, selective PL and IL influence into PAG and BLA	PTSD, anxiety
Manoocheri, 2021 [30]	Mice	Anterograde tracing, retrograde tracing, optogenetic stimulation, PFC layer-specific recordings	TC	rBLA and cBLA target distinct circuits within PL and IL cortex, rBLA and cBLA engage different networks that affect both PL and IL regions, leading to variations in threat processing and related behaviors	PTSD, anxiety
Shi, 2017 [56]	Rats	Quinpirole (D2 agonist) administration, sulpiride (D2 antagonist) administration, BLA protein analysis (GluR1/GluR1-Ser845/NR2B)	FC, ET, LTM, LAT	D2 activation enhances fear extinction, D2 blocking impairs fear extinction, changes in GluR1/GluR1-Ser845/NR2B proteins associated with these affects	Anxiety
Reppucci, 2016 [40]	Rats	mPFC and lateral hypothalamus retrograde tracer injections, BLA-mPFC connectivity mapping	CPP, LAT	BLA-mPFC disruption affects motivation and food-motivated behavior, BLA-mPFC projections show specific topographic organization	Anxiety
Arruda-Carvalho, 2017 [43]	Mice	AAV-ChR2 injection, optogenetic stimulation, mPFC-BLA pathway tracing		mPFC-BLA connections mature during adolescence, parallels emotional behavior maturation, it links to fear learning/regulation development	Anxiety, depression
Fucich, 2018 [49]	Rats	vmPFC DREADD injection, clozapine-N-oxide (CNO) administration, CUS protocol	SPDB, AST	Extinction reverses CUS induced deficits, vmPFC inhibition blocks extinction benefits, vmPFC activation restores normal behavior	PTSD
Saha, 2017 [39]	Rats	BLA lentiviral injections targeting neurofascin, AIS GABAergic input reduction	OFT, EPM, FC, FE	BLA neurofascin loss impairs fear extinction, no effect on anxiety behavior, no effect on fear acquisition/expression	Anxiety
Wang, 2021 [21]	Rats	CUMS depression protocol, mPFC/BLA LFP recordings, theta-gamma coupling analysis	SPT, FST, OFT	CUMS reduces exploration/movement, CUMS decreases time in central zone OFT, decreased mPFC theta-BLA gamma coupling	Depression
Printz, 2023 [23]	Mice	BLA rAAV2-retro-Cre injections, mPFC stCoChR/GCaMP6s AAV injection, optogenetic stimulation, calcium imaging, mPFC neuron patch-clamp recordings		mPFC-BLA neurons have distinct connectivity patterns that may influence processing within mPFC circuits, potentially impacting associated behaviors​	PTSD
Knox, 2016 [42]	Rats	c-Fos immunohistochemistry in vHipp/dHipp/BLA/PL/IL	SPS	SPS impairs extinction retention, animals show increased test freezing, it disrupts extinction circuits	PTSD
Liu, 2020 [41]	Mice	10-days CRS, optogenetic ChR2 PFC-BLA pathway activation, BLA neuron EPSC/IPSC recordings	EPM, OFT	CRS alters dmPFC-BLA E/I balance, increases BLA neuron excitation, links to elevated anxiety behavior	Anxiety, stress
Shelkar, 2021 [36]	Mice	AICP glycine agonist administration, GluN2C knockout mice, Astrocyte GluN1 conditional knockout, DREADD astrocyte activation, electrophysiological recordings	FC, FE, PERT	BLA astrocyte activation enhances extinction, reduces post-extinction freezing, CNO treatment strengthens extinction memory	Anxiety, PTSD
Han, 2023 [35]	Mice	Robotic predator exposure, mPFC/BLA LFP recordings, defense-related oscillation analysis	FFD, EGD	Animal show freeze/flight responses to robotic predator, linking to specific neuronal oscillations, mPFC-BLA circuit supports flexible switching between defensive behaviors depending on the threat context​	Anxiety
Qi, 2020 [52]	Rats	21-day CUMS protocol, Electrophysiological recordings	OFT, SPT, FST	Depression reduces mPFC-to-BLA information flow, BLA-to-mPFC flow remains normal, links to reduced exploration, mPFC-BLA disruption key in depression	Depression, anxiety
Hare, 2019 [54]	Mice	mPFC Drd1 neuron optogenetic stimulation, ketamine administration, cre-dependent DREADD inhibition	FST, EPM, NSF, SPT	mPFC Drd1 neuron activation reduces depression/anxiety, effects are rapid and sustained, Drd2 neuron activation shows no effect, Drd1 neurons crucial for mood regulation	Depression
Taub, 2018 [48]	Monkeys	BLA/dACC electrophysiology recording, theta oscillation analysis	CR	BLA-dACC theta increases during aversive learning, high synchrony during initial acquisition, synchrony decreases as learning plateaus	Anxiety, PTSD
Royer, 2002 [37]	Guinea pigs	BLA low-frequency stimulation for LTD, BLA high-frequency stimulation for LTP, electrophysiological recordings	LTP, LTD, PPF	Extinction creates competing new memory, ITC neurons show NMDA-dependent plasticity, LTP/LTD in ITC affects fear extinction, ITC controls BLA-CEA fear signaling	PTSD, Anxiety
Quirk, 2003 [38]	Rats and cats	mPFC electrical stimulation, electrophysiological recordings	AFC, ERT	mPFC stimulation reduces fear responses, decreases CEA neuron responsiveness, involves GABAergic intercalated cells, mPFC controls amygdala output	PTSD, GAD
